# Role of IL-1b in NLRP12-associated autoinflammatory disorders and resistance to anti-IL-1 therapy

**DOI:** 10.1186/1546-0096-9-S1-O31

**Published:** 2011-09-14

**Authors:** Isabelle Jeru, Véronique Hentgen, Sylvain Normand, Philippe Duquesnoy, Emmanuelle Cochet, Adriana Delwail, Gilles Grateau, Sandrine Marlin, Serge Amselem, Jean-Claude Lecron

**Affiliations:** 1 INSERM, Paris, France; 2 Centre Hospitalier de Versailles, Versailles, France; 3 Université de Poitiers, Poitiers, France; 4 Assistance Publique - Hôpitaux de Paris, Paris, France

## Background

A new class of autoinflammatory syndromes called *NLRP12*-associated disorders (NLRP12AD) has been associated with mutations in *NLRP12*. Conflicting data on the putative role of NLRP12 in IL-1b signaling have been generated *in vitro*.

## Aim

This prospective study was undertaken to assess the secretion of IL-1b and three IL-1b-induced cytokines (IL-1Ra, IL-6 and TNF-a) in patients’ PBMC cultured *ex vivo* and to evaluate the patients’ response to recombinant IL-1 receptor antagonist (IL-1Ra, anakinra), a major drug in the treatment of autoinflammatory disorders.

## Methods

Patients’ disease manifestations and cytokine measurements were recorded before anakinra treatment was started, during 14 months of therapy, and after discontinuation of anakinra treatment.

## Results

Spontaneous secretion of IL-1b by patients’ PBMC was found to be dramatically increased (80 to 175-fold) compared to controls. Consistently, anakinra initially led to a marked clinical improvement and to a rapid near-normalization of IL-1b secretion. However, a progressive clinical relapse occurred secondarily, associated with an increase in TNF-a secretion, persistent elevated levels of IL-1Ra and IL-6 and a reactivation of IL-1b secretion. Anakinra was discontinued after 14 months of therapy.

## Conclusion

Our findings provide *in vivo* evidence of the crucial role of IL-1b in the pathophysiology of NLRP12AD. This is the first time anakinra has been used to treat this disorder. This study provides new insights into the mechanisms underlying resistance to anti–IL-1 therapy observed in few patients with autoinflammatory syndromes. Our data also point to the potential interest of cytokine *ex vivo* measurements as predictors of response to treatment.

